# Neuropilin 1 balances β8 integrin-activated TGFβ signaling to control sprouting angiogenesis in the brain

**DOI:** 10.1242/dev.113746

**Published:** 2015-12-15

**Authors:** Shinya Hirota, Thomas P. Clements, Leung K. Tang, John E. Morales, Hye Shin Lee, S. Paul Oh, Gonzalo M. Rivera, Daniel S. Wagner, Joseph H. McCarty

**Affiliations:** 1 Department of Neurosurgery, University of Texas MD Anderson Cancer Center, Houston, TX 77030, USA; 2 Department of Biosciences, Rice University, Houston, TX 77005, USA; 3 College of Veterinary Medicine, Texas A&M University, College Station, TX 77843, USA; 4 Department of Physiology and Functional Genomics, University of Florida, Gainseville, FL 32610, USA

**Keywords:** Endothelial cell, Extracellular matrix, Neurovascular unit, *itgb8*, *nrp1*, *tgfbr2*

## Abstract

Angiogenesis in the developing central nervous system (CNS) is regulated by neuroepithelial cells, although the genes and pathways that couple these cells to blood vessels remain largely uncharacterized. Here, we have used biochemical, cell biological and molecular genetic approaches to demonstrate that β8 integrin (Itgb8) and neuropilin 1 (Nrp1) cooperatively promote CNS angiogenesis by mediating adhesion and signaling events between neuroepithelial cells and vascular endothelial cells. β8 integrin in the neuroepithelium promotes the activation of extracellular matrix (ECM)-bound latent transforming growth factor β (TGFβ) ligands and stimulates TGFβ receptor signaling in endothelial cells. Nrp1 in endothelial cells suppresses TGFβ activation and signaling by forming intercellular protein complexes with β8 integrin. Cell type-specific ablation of β8 integrin, Nrp1, or canonical TGFβ receptors results in pathological angiogenesis caused by defective neuroepithelial cell-endothelial cell adhesion and imbalances in canonical TGFβ signaling. Collectively, these data identify a paracrine signaling pathway that links the neuroepithelium to blood vessels and precisely balances TGFβ signaling during cerebral angiogenesis.

## Abstract (precis)

**Summary:** Neuropilin 1 and β8 integrin cooperatively promote cerebral angiogenesis by mediating adhesion and signaling events between neuroepithelial cells and vascular endothelial cells in the mouse brain.

## INTRODUCTION

During CNS development, neuroepithelial cells interact with angiogenic blood vessels via ECM-rich vascular basement membranes to modulate patterns of endothelial cell growth and sprouting (Engelhardt and Sorokin, 2009). Integrins are receptors for many ECM protein ligands (Kim et al., 2011), and integrin-mediated adhesion and signaling pathways promote CNS vascular development and homeostasis (del Zoppo and Milner, 2006; McCarty, 2009). In particular, the neuroepithelial-expressed αvβ8 integrin and its ECM protein ligands, the latent TGFβs, are key regulators of angiogenesis in the developing CNS (McCarty et al., 2005b, 2002; Proctor et al., 2005; Zhu et al., 2002). Cells produce TGFβs as latent, inactive complexes that are sequestered in the ECM prior to activation (Worthington et al., 2011). αvβ8 integrin adheres to RGD sequences within the latency-associated protein (LAP) of TGFβs and mediates cytokine release from the ECM and activation of TGFβ receptor signaling pathways (Allinson et al., 2012; Arnold et al., 2012; Cambier et al., 2005; Hirota et al., 2011). Point mutations in latent TGFβ1 that inhibit integrin binding lead to developmental defects that phenocopy those in *Tgfb1*^−/−^ mice (Yang et al., 2007). Combined loss of TGFβ1 and TGFβ3 activation lead to brain angiogenesis pathologies that phenocopy those in αv and β8 integrin mutant mice (Mu et al., 2008), highlighting the *in vivo* significance of integrin control of TGFβ activation and signaling. We have shown, using Cre-lox mouse models, that ablation of TGFβR2 or Alk5 (also known as TGFβR1) in endothelial cells, but not neuroepithelial cells, results in brain vascular pathologies that are similar to phenotypes that develop in β8 integrin and TGFβ1/3 mutant mice (Nguyen et al., 2011). TGFβ receptors phosphorylate various intracellular signaling effectors, including Smad transcription factors (Massagué, 2012). Genetic deletion of Smad4 in endothelial cells leads to angiogenesis defects and intracerebral hemorrhage, revealing that canonical TGFβ receptor signaling is essential for normal brain vascular development (Li et al., 2011). Proteins that negatively regulate αvβ8 integrin-mediated activation of latent TGFβs and subsequent TGFβ signaling have remained largely unknown.

Nrp1 is a 130 kDa transmembrane protein expressed in endothelial cells as well as some neurons and glia (Eichmann et al., 2005). Nrp1 is a receptor for multiple ligands including semaphorins (He and Tessier-Lavigne, 1997), vascular endothelial growth factor-A (Vegfa) (Soker et al., 1998), hepatocyte growth factor (Hu et al., 2007), and hedgehog proteins (Hillman et al., 2011). Mice genetically null for Nrp1 in all cells develop vascular pathologies including impaired cerebral angiogenesis and die embryonically (Gerhardt et al., 2004). Selective ablation of Nrp1 in endothelial cells leads to angiogenic sprouting defects (Gu et al., 2003) that occur independently of semaphorins (Gu et al., 2005), suggesting that impaired Nrp1 binding to Vegfa is the primary defect. However, genetic ablation of Vegfa in the neuroepithelium does not phenocopy the vascular defects in Nrp1 mutant mice (Haigh et al., 2003), and antibody-mediated inhibition of Nrp1-Vegfa interactions does not block angiogenesis (Pan et al., 2007). Genetic ablation of Nrp1 in neuroepithelial cells or macrophages does not lead to developmental vascular pathologies (Fantin et al., 2013). Furthermore, mice expressing an engineered point mutation in the Nrp1 extracellular region (Y297A) that abrogates Vegfa binding do not develop obvious brain pathologies (Fantin et al., 2014). Hence, the mechanisms by which Nrp1 in endothelial cells controls cerebral angiogenesis independently of Vegfa and semaphorin signaling remain enigmatic.

Here, we have generated and analyzed various mouse and zebrafish mutant models to demonstrate that Nrp1 and β8 integrin cooperatively regulate cerebral angiogenesis. Paracrine interactions between β8 integrin and Nrp1 couple the neuroepithelium to blood vessels and balance TGFβ signaling via Smad family members in the endothelium. Mice lacking any component of the β8 integrin-Nrp1-TGFβ signaling pathway develop brain vascular pathologies, including impaired sprouting angiogenesis and hemorrhage. Collectively, these results identify novel components of an adhesion and signaling axis that couples neuroepithelial cells and endothelial cells to fine-tune sprouting angiogenesis during embryonic brain development.

## RESULTS

**Fig. 1.**
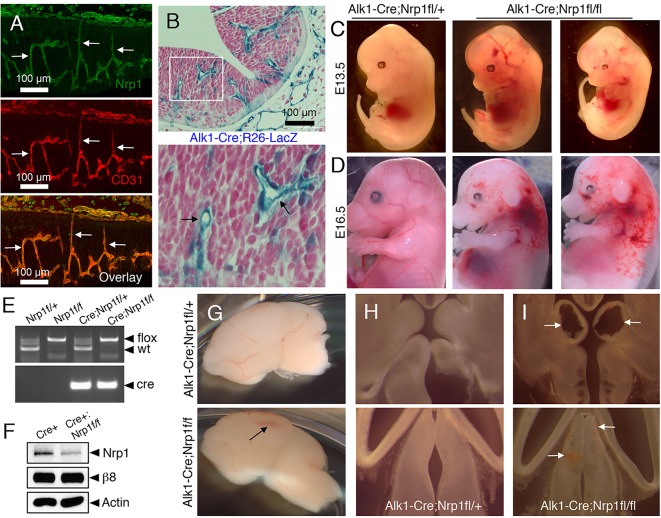
**Genetic ablation of Nrp1 in endothelial cells leads to brain vascular pathologies and embryonic lethality.** (A) E13.5 horizontal brain sections were labeled with anti-Nrp1 (green) and anti-CD31 (red) antibodies. Note that Nrp1 protein is expressed at robust levels in endothelial cells as revealed by co-localization with CD31 (arrows). (B) *Alk1-Cre* knock-in mice were crossed to the *Rosa26-loxSTOPlox-lacZ* reporter strain and E10.5 brain sections were stained with X-Gal (blue) and Hematoxylin (red). The *Alk1* promoter drives Cre expression primarily in cerebral blood vessels (arrows in lower panel). (C,D) *Alk1-Cre* mice were crossed to mice harboring a conditional floxed *Nrp1* gene (*Nrp1^fl/fl^*). Control (left panels) and mutant (right panels) embryos were analyzed at E13.5 (C) and E16.5 (D), revealing edema and hemorrhage in *Alk1-Cre;Nrp1^fl/fl^* mutants. (E) Genotypes of embryos at E13.5 as identified by genomic PCR. (F) Immunoblots of brain lysates from control and *Alk1-Cre;Nrp1^fl/fl^* embryos. Residual Nrp1 protein levels are likely a result of expression in the neuroepithelium. (G) Brains were dissected from E14.5 control (top) and mutant (bottom) embryos. Note the focal area of hemorrhage in the mutant brain (arrow). (H,I) Horizontal sections through brains of *Alk1-Cre* (H), or *Alk1-Cre;Nrp1^fl/fl^* (I) embryos, with arrows revealing cavitations and punctate microhemorrhage within the ganglionic eminences (upper panel) and thalamus (lower panel) of mutant brains (I).

We analyzed spatial patterns of Nrp1 protein expression in the developing mouse brain by labeling embryonic sections with antibodies that recognize the Nrp1 extracellular domain. Nrp1 protein was expressed in brain endothelial cells (Fig. 1A), with lower levels of Nrp1 protein detected in neuroepithelial cells (Fig. S1A), which is consistent with published reports (Fantin et al., 2013). Because whole body deletion of Nrp1 results in embryonic lethality by embryonic day (E) 11 (Kawasaki et al., 1999), we selectively ablated Nrp1 using an engineered mouse model in which the endogenous Alk1 (also known as Acvrl1) promoter drives expression of Cre in vascular endothelial cells (Nguyen et al., 2011). The *Alk1* gene encodes a type 1 receptor for members of the TGFβ superfamily that is expressed in endothelial cells during development (Park et al., 2008). *Alk1*-Cre is active at early stages of brain angiogenesis, as revealed by intercrosses with the *Rosa26-loxSTOPlox-lacZ* reporter strain (Fig. 1B). Compared with other endothelial promoters such as *Tie1* or *Tie2*, the *Alk1* promoter drives Cre expression in the developing yolk sac vasculature 24 to 48 h later in development (Nguyen et al., 2011). This temporal expression of Cre via the *Alk1* promoter is crucial, as requirements for genes in yolk sac angiogenesis are largely circumvented. For example, genetic ablation of the murine gene encoding TGFβR2 (*Tgfbr2*) using *Tie1-Cre* leads to lethality by E10.5 resulting from heart and yolk sac vascular defects (Carvalho et al., 2007). In contrast, *Alk1-Cre* deletion of *Tgfbr2* allows for survival until E15 (Nguyen et al., 2011), providing an opportunity to analyze related signaling pathways in brain vascular development.

*Alk1-Cre/+;Nrp1^fl/+^* male mice were bred to *Nrp1^fl/fl^* females to generate control (*Alk1-Cre/+;Nrp1^fl/+^*) or mutant (*Alk1-Cre/+;Nrp1^fl/fl^*) progeny. Genotyping of newborn mice [*n*=27 postnatal day (P) 0 mice from six different litters] revealed no viable *Alk1-Cre/+;Nrp1^fl/fl^* mutant pups. Therefore, we analyzed embryos at E11.5, E13.5 and E16.5. Expected Mendelian ratios of control and knockout embryos were found at E11.5 (*n*=33 embryos, 9 viable mutants or 27%) and E13.5 (*n*=27 embryos, 6 viable mutants or 22%). All E13.5 mutant embryos were viable and appeared developmentally normal, although some knockouts displayed microhemorrhages in the head and body (Fig. 1C). By contrast, *Alk1-Cre/+;Nrp1^fl/fl^* mutants at E16.5 (*n*=3 embryos) were growth-impaired and displayed widespread edema and hemorrhage (Fig. 1D). Two non-viable mutants were discovered at E16.5 that showed extensive necrosis (data not shown). All genotypes were confirmed by PCR with genomic DNA isolated from tissue snips (Fig. 1E). Immunoblots of brain lysates from mutant animals showed a significant reduction in total Nrp1 protein (Fig. 1F). Unlike controls, all *Alk1-Cre/+;Nrp1^fl/fl^* conditional mutant embryos analyzed displayed focal regions of brain hemorrhage (Fig. 1G). More detailed analyses of brain sections revealed cavitations and areas of hemorrhage primarily within the developing ganglionic eminences and thalamus (Fig. 1H-I).

Vascular pathologies in Nrp1 conditional knockouts appeared strikingly similar to phenotypes that have been reported in mice lacking αv or β8 integrin in the neuroepithelium (McCarty et al., 2005b; Proctor et al., 2005). Indeed, side-by-side comparisons of brains from *Alk1-Cre/+;Nrp1^fl/fl^* mutants, with *Nestin-Cre;β8^fl/fl^* and β8 integrin null (*β8^−/−^*) mutants revealed similar pathologies within the ganglionic eminences and thalamus (Fig. S1B-C). Blood vessel patterning defects and hemorrhage were detected in mouse embryos lacking αv integrin in the neuroepithelium via *Nestin-Cre* (Fig. S2), revealing that specific loss of the αvβ8 integrin heterodimer in the neuroepithelium contributes to these vascular defects.

**Fig. 2.**
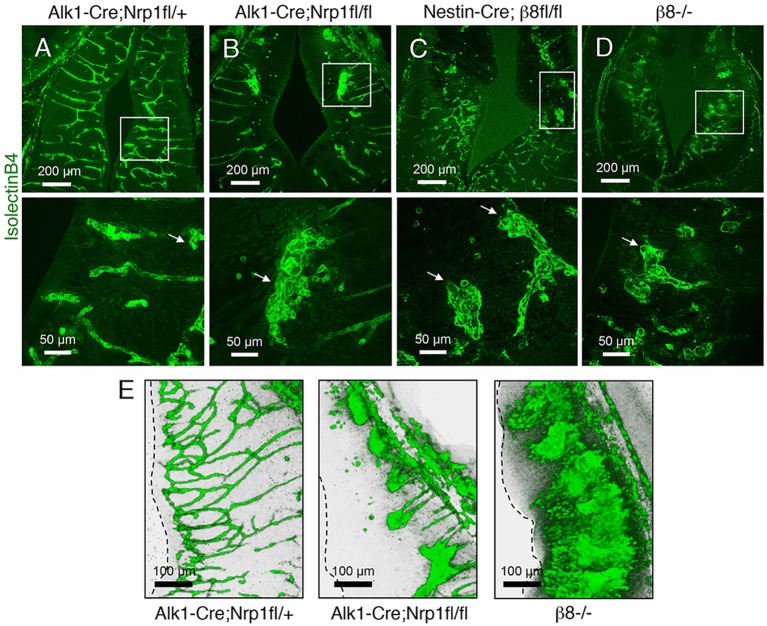
**Analysis of brain vascular pathologies in mice lacking Nrp1 in endothelial cells or β8 integrin in neuroepithelial cells.** (A-D) Horizontal sections through the ganglionic eminences of control (A), *Alk1-Cre;Nrp1^fl/fl^* (B), *Nestin-Cre;β8^fl/fl^* (C), or *β8^−/−^* (D) embryos labeled with Isolectin B4-Alexa Fluor 488 to reveal blood vessels. Lower panels are digitally magnified images of boxed areas in upper panels. Note the abnormal blood vessel patterning in the mutant brains (arrows). (E) Horizontal sections through the thalamus of control (left), *Alk1-Cre;Nrp1^fl/fl^* (middle) and *β8^−/−^* (right) E13.5 brains were labeled with anti-CD31 antibodies. Shown are representative three-dimensional reconstructions of the brain vasculature. At this developmental age, note that Nrp1 mutant blood vessels fail to sprout normally and do not reach the subventricular zone (dashed line), whereas blood vessels in the *β8^−/−^* brain display abnormal hyper sprouting near subventricular regions.

We next analyzed microscopic blood vessel morphologies in control and mutant mice by labeling brain slices with fluorescently conjugated Isolectin B4 to visualize vascular endothelial cells. Blood vessels in control embryos showed radial patterns of invasion throughout the brain parenchyma (Fig. 2A). By contrast, blood vessels in Nrp1 and β8 integrin mutant brains showed aberrant patterning and formed glomeruloid-like tufts, as well as hemorrhage (Fig. 2B-D). Interestingly, in *Alk1-Cre/+;Nrp1^fl/fl^* conditional knockout embryonic brains we detected blood vessels that failed to properly sprout and form more elaborate networks near the subventricular zone. By contrast, sprouting blood vessels in *β8^−/−^* embryos reached subventricular regions but formed abnormal glomeruloid-like tufts (Fig. 2E; Fig. S3), which is consistent with a prior study showing hyperactive angiogenic sprouting in β8 integrin mutant brains (Arnold et al., 2014). To determine if the phenotypes in *β8^−/−^* mice were linked to integrin control of Nrp1 protein expression, control and *β8^−/−^* brain sections were immunolabeled with anti-Nrp1 antibodies. Nrp1 protein was expressed at comparable levels in cerebral blood vessels of control and *β8^−/−^* embryos (Fig. S4A,B). By contrast, Nrp1 protein was absent in cerebral blood vessels in *Alk1-Cre/+;Nrp1^fl/fl^* mutant mice owing to gene ablation (Fig. S4C). Similarly, Nrp1 protein was expressed in detergent-soluble brain lysates from control and *β8^−/−^* mutant embryos (Fig. S4D).

Pericytes are essential for cerebral angiogenesis and endothelial barrier formation (Armulik et al., 2010; Daneman et al., 2010), which prompted us to determine if vascular pericytes were absent in Nrp1 conditional knockout mice. Immunofluorescence with anti-NG2 antibodies revealed that endothelial cells were associated with pericytes in control as well as *Alk1-Cre/+;Nrp1^fl/fl^* and *β8^−/−^* mutant mice (Fig. S5). Similar results were found with an antibody targeting the pericyte-enriched protein desmin (data not shown). Analysis of murine gene expression databases revealed that *Itgb8* mRNA is expressed primarily in the embryonic neuroepithelium (Fig. S6A). *Nrp1* showed a broader pattern of expression, although within the brain parenchyma *Nrp1* mRNA was present most notably in blood vessels (Fig. S6B-C). Immunofluorescence labeling of brain sections revealed αv integrin protein expression in the neuroepithelium and Nrp1 expression in blood vessels, with co-localization at points of neuroepithelial-blood vessel contacts (Fig. S6D). Consistent with these *in vivo* expression patterns, we have shown previously that β8 integrin, which dimerizes exclusively with the αv subunit, is expressed in cultured neuroepithelial cells (Mobley et al., 2009). In addition, the immortalized mouse brain endothelial cell line bEND.3 (Montesano et al., 1990) and primary endothelial cells isolated from the human umbilical vein (HUVECs) expressed robust levels of Nrp1 protein (Fig. S7).

**Fig. 3.**
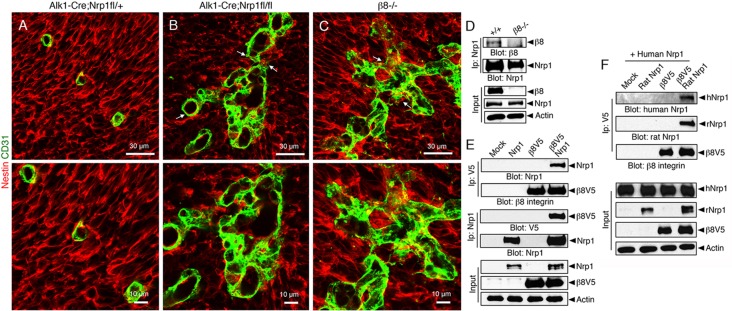
**β8 integrin and Nrp1 form protein complexes and promote neuroepithelial-endothelial cell adhesion.** (A-C) E13.5 control (A) and mutant (B,C) brain sections were immunostained with anti-CD31 (green) and anti-Nestin antibodies (red) to visualize endothelial cells and neuroepithelial cells, respectively. Note the defective cell-cell interactions and disorganized patterns of perivascular neuroepithelial cells in mutant samples (arrows in B,C upper panels). (D) Nrp1 and β8 integrin proteins co-immunoprecipitate in detergent-soluble protein lysates from wild-type neonatal mouse brains. By contrast, protein-protein interactions are not detected in *β8^−/−^* brain lysates. (E) HEK-293 cells were transfected with plasmids expressing full-length rat Nrp1 and human β8 integrin containing a V5 epitope tag at the C-terminus. Detergent-soluble lysates were immunoprecipitated with anti-V5 antibodies and immunoblotted with anti-Nrp1 antibodies. Note that β8 integrin and Nrp1 protein complexes are detected only in cells forcibly expressing both proteins. (F) Cells expressing human NRP1 were mixed with cells expressing rat Nrp1, V5-tagged human β8 integrin, or rat Nrp1 and human V5-tagged β8 integrin in combination. Detergent-soluble lysates were immunoprecipitated with anti-V5 antibodies and then immunoblotted with species-specific anti-Nrp1 antibodies to distinguish binding with human NRP1 (trans) or rat Nrp1 (cis and trans). Note that β8 integrin and human NRP1 interact in trans, but only when rat Nrp1 is co-expressed with β8 integrin.

We hypothesized that the similar brain vascular pathologies in Nrp1 and β8 integrin mutant mice were a result of defective adhesion and signaling between Nrp1 in endothelial cells and αvβ8 integrin in the neuroepithelium. Therefore, we performed immunofluorescence experiments to visualize interactions between blood vessels and neuroepithelial cells. Cerebral blood vessels in control brains showed close juxtaposition with the surrounding neuroepithelium (Fig. 3A). By contrast, neuroepithelial cells in *Alk1-Cre/+;Nrp1^fl/fl^* and *β8^−/−^* brains did not closely juxtapose blood vessels (Fig. 3B,C) and appeared fragmented, especially at perivascular contact points. Interactions between Nrp1 and β8 integrin were found in protein complexes in wild-type mouse brain lysates, as revealed by co-immunoprecipitation (Fig. 3D). We also analyzed protein-protein interactions using *in vitro* assays. Protein complexes were detected in HEK-293 cells transiently expressing V5-tagged human β8 integrin or full-length rat Nrp1 (Fig. 3E). These immunoprecipitation experiments did not discern whether Nrp1 and β8 integrin proteins interact via mechanisms involving cis (the same cell) or trans (different cells) binding. Therefore, we analyzed Nrp1-β8 integrin interactions in cells expressing each protein alone or in different combinations. When cells expressing human NRP1 were mixed with cells expressing β8 integrin we did not detect protein-protein interactions by co-immunoprecipitation. However, when rat Nrp1 was co-expressed with β8 integrin, trans interactions between β8 integrin and human NRP1 were detected using species-specific anti-Nrp1 antibodies (Fig. 3F). These data reveal that Nrp1 in adjacent cell types is important for the formation of trans Nrp1-β8 integrin protein complexes. These *in vitro* results support our *in vivo* data showing that Nrp1 is expressed in endothelial cells and closely juxtaposed neuroepithelial cells (Fig. 1; Fig. S1), whereas αvβ8 integrin is expressed only in neuroepithelial cells (Fig. S6). These results are also consistent with a prior report showing that Nrp1 can signal via both cis and trans mechanisms (Koch et al., 2014).

To identify Nrp1 domains that mediate binding to β8 integrin we generated various Nrp1 deletion constructs lacking the cytoplasmic tail or different extracellular domains involved in dimerization or ligand binding (Fig. S8A). However, deletion of the entire Nrp1 cytoplasmic tail or various extracellular domains (A, B and MAM domains) did not block binding to β8 integrin (Fig. S8B-D), suggesting the involvement of more than one Nrp1 domain in mediating integrin interactions. Using transfection strategies in HEK-293T cells, we also detected protein complexes containing Nrp1 and TGFβR2, which is consistent with a recent report showing that Nrp1 suppresses TGFβ receptor signaling in sprouting endothelial cells (Aspalter et al., 2015). These interactions could not be blocked by deletion of the Nrp1 cytoplasmic domain or various extracellular domains (Fig. S8E-G).

**Fig. 4.**
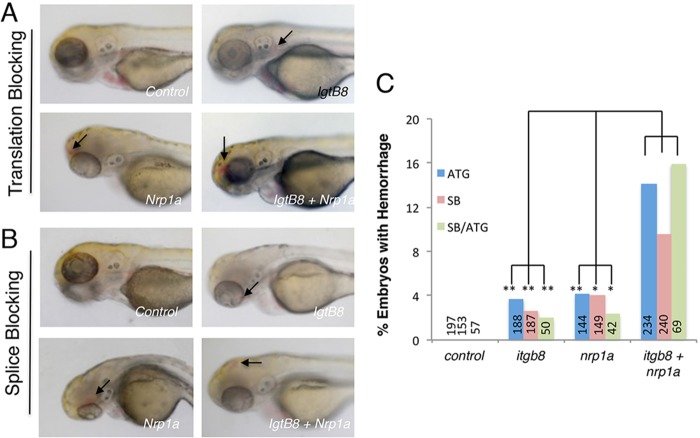
***itgb8* and *nrp1a* genetically interact to promote normal brain vascular development in zebrafish.** (A,B) Zebrafish embryos injected with control MOs or MOs designed to block *itgb8* or *nrp1a* translation (A) or splicing (B). No hemorrhage or other vascular defects were obvious at 3 days post-fertilization in embryos injected with control MOs. However, hemorrhage (arrows) is observed in the heads of fish injected with *itgb8* or *nrp1a* translation or splice blocking MOs. In addition, double injection of *itgb8* and *nrp1a* MOs leads to a higher incidence of cranial hemorrhage (arrows in lower right panels). (C) Quantitation of cerebral hemorrhage phenotypes in embryos injected with single translation blocking (ATG) MOs, splice blocking MOs, or both MOs injected in combination. Numbers of embryos analyzed for each MO are indicated. Translation blocking *itgb8* versus *itgb8/nrp1a*, *P*=0.0003; translation blocking *nrp1a* versus *nrp1a/itgb8*, *P*=0.002; splice blocking *itgb8* versus *itgb8/nrp1a*, *P*=0.004; splice blocking *nrp1a* versus *nrp1a/itgb8*, *P*=0.04.

If Nrp1 and β8 integrin interact physically we expected that they would also display a genetic interaction. A decrease in expression of both genes should reveal a phenotype, whereas decreasing expression of either gene individually will not. However, revealing this interaction might require decreasing the level of each below that found in heterozygotes for either gene. Indeed, Nrp1/β8 integrin double heterozygotes, which express 50% of each gene product, do not display obvious brain vascular defects (data not shown). To further investigate genetic interactions we used the zebrafish *Danio rerio*, which contains a neurovascular unit cytoarchitecture that is structurally and functionally similar to mammals (Ulrich et al., 2011). Zebrafish are also amenable to the use of morpholino antisense oligonucleotides (MOs), which repress the expression of genes by directly blocking translation and/or splicing. This technology allows us to titrate single doses of MOs to the lowest effective level required to observe phenotypes, and then test the effects of combinations of MOs. Injection of low amounts of either translation blocking (Fig. 4A) or splice blocking (Fig. 4B) MOs targeting *itgb8* or *nrp1a* resulted in a rate of cranial hemorrhage of 2-4%. Injection of both MOs in combination resulted in a significant increase in cranial hemorrhage to a rate up to 16% (Fig. 4C). The rate observed for the double injections is larger than the sum of the single injections, suggesting synergy in the genetic interaction (Fig. 4D). The efficacy of the control and targeting MOs was tested by PCR spanning the affected intron, revealing a nearly 50% reduction in *itgb8* expression and a complete loss of *nrp1a* expression (data not shown).

**Fig. 5.**
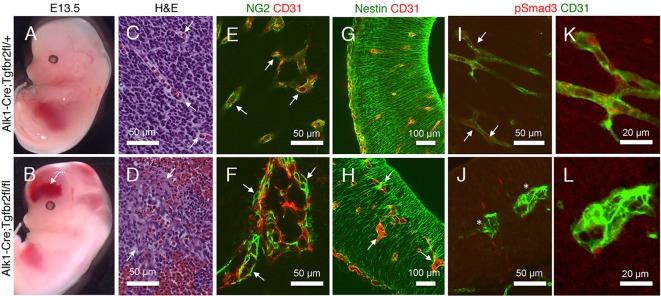
**TGFβ signaling in endothelial cells is essential for brain vascular development.** (A,B) *Alk1-Cre/+* control (A) and *Alk1-Cre/+;Tgfbr2^fl/fl^* mutant (B) embryos were analyzed at E13.5, revealing severe intracerebral hemorrhage in conditional knockouts (arrow in B). (C,D) Horizontal sections through the ganglionic eminences of E13.5 control (C) and mutant (D) embryos were stained with H&E, revealing hemorrhage and blood vessel patterning defects in mutant brains (arrows in D). (E,F) Control (E) and mutant (F) brain sections were immunostained with anti-CD31 and anti-NG2 antibodies. Note that mutant blood vessels display glomeruloid-like morphologies but contain pericytes (arrows in F). (G,H) Control (G) and mutant (H) brain sections were labeled with anti-CD31 (red) and anti-Nestin (green) antibodies, revealing aberrant contacts between endothelial cells and surrounding neuroepithelial cells (arrows in H). (I,J) *Alk1-Cre* (I) and mutant (J) brain sections were labeled with anti-CD31 and anti-pSmad3 antibodies. Note the diminished Smad3 activation in mutant endothelial cells (asterisks in J). (K,L) Higher magnification images from panels I and J, respectively, showing diminished levels of phosphorylated Smad3 within endothelial nuclei in mutant brains. Arrows in C,E,I indicate the wild-type condition for comparison with mutant abnormalities in D,F and J, respectively.

αvβ8 integrin controls angiogenesis by triggering activation of ECM-bound latent TGFβs and stimulating TGFβ receptor intracellular signaling in endothelial cells (Arnold et al., 2012; Hirota et al., 2011). To study potential links between Nrp1 and the TGFβ signaling pathway during angiogenesis, we interbred *Alk1-Cre* mice with mice harboring a conditional *Tgfbr2* gene (*Tgfbr2^fl/fl^*) (Chytil et al., 2002) to generate control (*Alk1-Cre*) and mutant (*Alk1-Cre;Tgfbr2^fl/fl^*) embryos. *Alk1-Cre;Tgfbr2^fl/fl^* mutant mice developed massive intracerebral hemorrhage (Fig. 5A-D), and no viable embryos were found beyond E16 as we have reported previously (Nguyen et al., 2011). The brain vascular pathologies in *Tgfbr2* mutants were not a result of loss of blood vessel-associated pericytes (Fig. 5E,F), but did correlate with defective adhesion between endothelial cells and the surrounding neuroepithelium (Fig. 5G,H). *Alk1-Cre;Tgfbr2^fl/fl^* mutant endothelial cells within the ganglionic eminences and thalamus contained less phosphorylated Smad3 (pSer423/425) protein (Fig. 5I-L). Tgfbr2 mutant mice did not show diminished Nrp1 protein levels in blood vessels (Fig. S7), and differences in β8 integrin protein expression were not detected in *Alk1-Cre;Nrp1^fl/fl^* brain lysates (Fig. 1F).

**Fig. 6.**
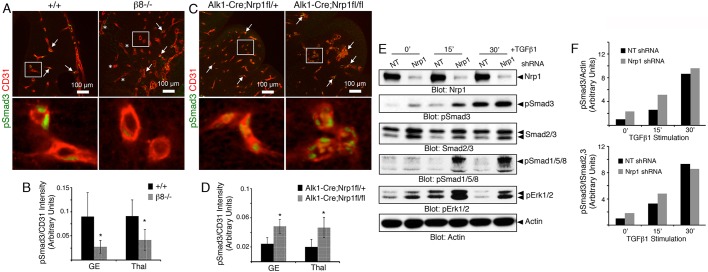
**Nrp1 and β8 integrin cooperatively balance TGFβ signaling in brain endothelial cells.** (A) Horizontal sections through the cerebral cortices of E12.5 wild-type and *β8^−/−^* embryonic brains were immunostained with anti-pSmad3 and anti-CD31 antibodies to visualize canonical TGFβ signaling in endothelial cells. Arrows indicate blood vessels containing nuclear pSmad3, whereas asterisks denote blood vessels lacking pSmad3. Lower panels are digitally magnified images of boxed areas in upper panels. (B) Quantitation of phosphorylated Smad3 levels in CD31^+^ endothelial cells within control and mutant cortical regions. Note the reduction in Smad3 phosphorylation in the *β8^−/−^* brain samples, *P<0.05, error bars represent s.d. (C) Horizontal brain sections from E13.5 *Alk1-Cre* control and *Alk1-Cre;Nrp1^fl/fl^* mutant embryos were immunostained with anti-pSmad3 and anti-CD31 to visualize TGFβ signaling in endothelial cells. Arrows indicate blood vessels containing nuclear pSmad3. Lower panels are higher magnification images of boxed areas in upper panels. (D) Quantitation of phosphorylated Smad3 levels in CD31^+^ endothelial cells within control and mutant cortical brain regions. Note that endothelial cells lacking Nrp1 contain significantly elevated levels of phosphorylated Smad3, *P<0.05, error bars represent s.d. (E) Endothelial cells infected with lentiviruses expressing GFP as well as non-targeting (NT) or *Nrp1* shRNAs were stimulated with TGFβ1 for varying times and lysates were immunoblotted with the indicated antibodies. Note the higher levels of pSmad3, pSmad1/5/8 and pErk1/2 at baseline and following TGFβ1 stimulation. Nrp1-dependent differences in phosphorylated Akt1 or p38α were not detected. (F) Quantitation of Nrp1-dependent Smad3 phosphorylation levels before and after TGFβ1 stimulation based on the representative immunoblot in E, plotted as pSmad3 levels normalized to actin (upper graph) or normalized to total Smad2/3 (lower graph). GE, ganglionic eminences; Thal, thalamus.

To further link Nrp1 and β8 integrin to TGFβ signaling *in vivo*, we labeled brain sections from control and mutant embryos with antibodies recognizing phosphorylated Smad3 and CD31 (also known as Pecam1), respectively. We focused on blood vessels within the developing ganglionic eminences and thalamus, where angiogenesis defects were evident but severe hemorrhage was absent. Phosphorylated Smad3 protein was detected in endothelial cells of control cerebral blood vessels. A significant decrease in Smad3 phosphorylation in endothelial cells was detected in *β8^−/−^* brains (Fig. 6A,B), similar to the lower levels in *Alk1-Cre;Tgfbr2^fl/fl^* mutant embryos (Fig. 5). By contrast, *Alk1-Cre;Nrp1^fl/fl^* knockout brains showed three-fold higher levels of pSmad3 in endothelial cells (Fig. 6C,D). A similar increase in pSmad1/5/8 levels was detected in cerebral endothelial cells in *Alk1-Cre;Nrp1^fl/fl^* mutant embryos (Fig. S9). In support of the *in vivo* data, silencing *Nrp1* gene expression in cultured endothelial cells using lentiviral-expressed shRNAs caused significantly enhanced baseline levels of phosphorylated Smad3 and Smad1/5/8. Addition of TGFβ1 to cells expressing *Nrp1* shRNAs led to higher levels of Smad phosphorylation in comparison to controls (Fig. 6E,F). A similar increase in phosphorylation of Erk1 and Erk2 (also known as Mapk3 and Mapk1, respectively) was detected (Fig. 6E), revealing that Nrp1 suppresses Smad-dependent and Smad-independent signaling events.

**Fig. 7.**
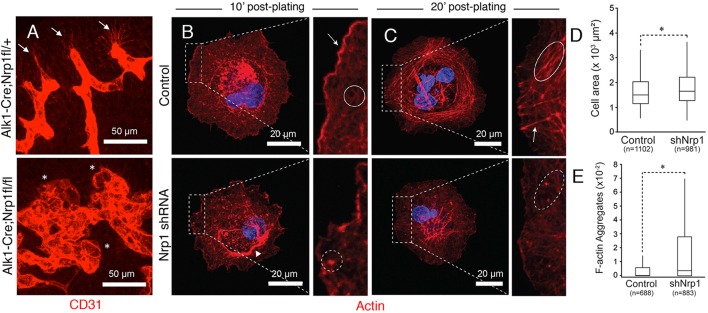
**Nrp1 controls F-actin dynamics in endothelial cells.** (A) Horizontal brain sections through E13.5 ganglionic eminences from *Alk1-Cre* control (top panel) or *Alk1-Cre;Nrp1^fl/fl^* mutants (lower panel) were immunolabeled with anti-CD31. Note the polarized endothelial tip cell filopodia in control brains (arrows). By contrast, Nrp1 mutant brains show defective tip cell sprouting and form glomeruloid-like tufts (asterisks). (B,C) Endothelial cells expressing NT shRNAs (upper panels) or *Nrp1* shRNAs (lower panels) were plated on fibronectin, allowed to spread for 10 min (B) or 20 min (C) and labeled with Phalloidin-Texas Red to visualize the actin cytoskeleton. Cells expressing NT shRNAs form an elaborate cortical actin network at 10 min and transverse actin arcs at 20 min, whereas cells expressing *Nrp1* shRNAs display abnormalities in the cortical actin network and instead form F-actin aggregates. Arrows show actin arcs. (D,E) Quantitation of endothelial cell spreading at 20 min post-adhesion (D), and actin aggregate formation at 10 min post-adhesion (E). Cells expressing *Nrp1* shRNAs show subtle, but statistically significant, increases in spreading, and more obvious defects in organization of the F-actin network. Total numbers of endothelial cells analyzed (*n*) are indicated, *P<0.05.

**Fig. 8.**
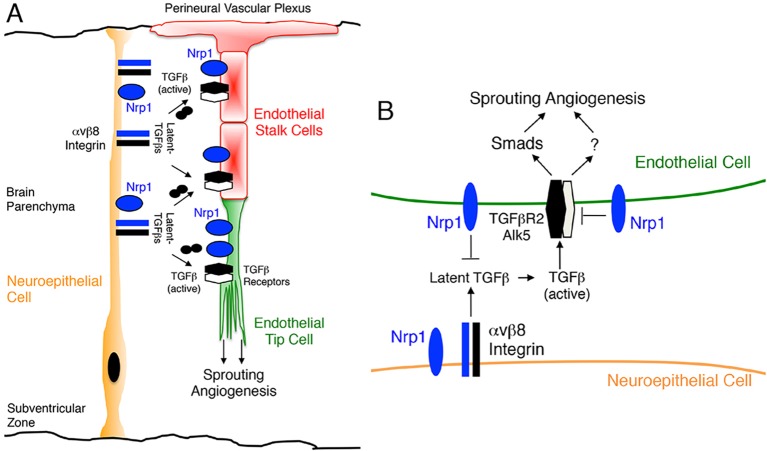
**Sprouting angiogenesis in the developing brain is coordinately regulated by the β8 integrin-TGFβ-Nrp1 signaling axis.** (A) αvβ8 integrin is expressed in the neuroepithelium where it controls angiogenesis by interacting with latent TGFβs in the ECM and Nrp1 in sprouting endothelial cells. Nrp1 is also expressed at low levels in neuroepithelial cells, and our data reveal that it promotes trans interactions between αvβ8 integrin and Nrp1 in endothelial cells. (B) Intercellular protein complexes between αvβ8 integrin and Nrp1 promote neuroepithelial-endothelial cell adhesion and modulate latent TGFβ activation and signaling. Genetic ablation of β8 integrin in neuroepithelial cells or TGFβR2 in endothelial cells inhibits the initial steps in the latent TGFβ activation and signaling cascade, leading to diminished Smad phosphorylation in endothelial cells. Deletion of Nrp1 in endothelial cells prevents normal suppression of αvβ8 integrin-mediated latent TGFβ activation and signaling, leading to elevated Smad phosphorylation. These imbalances in canonical TGFβ signaling in endothelial cells result in sprouting angiogenesis defects and intracerebral hemorrhage during development.

Endothelial tip cells are essential for normal sprouting angiogenesis and blood vessel patterning, and Nrp1 protein is enriched in these cells (Fantin et al., 2013). *Alk1-Cre;Nrp1^fl/fl^* mutant mice displayed defects in endothelial tip cell polarity, with blood vessels forming glomeruloid-like tufts with reduced numbers of filopodia (Fig. 7A). Actin cytoskeletal dynamics, particularly within endothelial tip cell filopodia, are important for sprouting angiogenesis (Gerhardt et al., 2003). Therefore, we next analyzed how loss of Nrp1 impacts the actin cytoskeleton in cultured endothelial cells. When endothelial cells expressing *Nrp1* shRNAs were plated on ECM, we detected defects in cell spreading and organization of the F-actin network (Fig. 7B-D). Unlike control cells (Movie 1), cells expressing *Nrp1* shRNAs exhibited faster spreading when compared with control cells. *Nrp1* shRNA cells showed poorly developed lamellipodia, presenting irregular edges that lacked active actin polymerization in the periphery (Movie 2). Furthermore, the presence of actin aggregates rather than incipient actin fibers was observed in the lamella of endothelial cells lacking Nrp1. These actin aggregates appeared to collapse into ring-like structures in the perinuclear region. At later time points the actin filaments initially observed in the lamella bundled into transverse arcs in control cells (Movie 3). Perpendicular actin fibers resembling stress fibers anchoring the cytoskeleton to sites of cell-substrate adhesion were also clearly distinguishable. By contrast, Nrp1-silenced cells showed a collapsed cytoskeleton with the presence of F-actin aggregates throughout the cell body and shorter, poorly organized actin bundles (Movie 4). Endothelial cells expressing *Nrp1* shRNAs did not show apparent defects in proliferation (data not shown) or formation of focal adhesions (Fig. S10). These data, showing Nrp1 functions in cultured endothelial cells, combined with our molecular genetic and biochemical results, reveal that the αvβ8 integrin-Nrp1 adhesion pathway balances TGFβ signaling to control proper sprouting angiogenesis. Targeting any component in this paracrine axis leads to cell adhesion and sprouting defects resulting in similar brain vascular pathologies (Fig. 8).

## DISCUSSION

Here we report a new cell adhesion and signaling pathway comprising Nrp1 in endothelial cells and αvβ8 integrin in neuroepithelial cells that precisely controls sprouting angiogenesis in the brain. Specifically, our experiments reveal the following novel findings: (i) genetic ablation of Nrp1 in vascular endothelial cells via *Alk1-Cre* leads to embryonic lethality associated with defective sprouting angiogenesis and hemorrhage (Fig. 1); (ii) brain vascular pathologies in *Alk1-Cre;Nrp1^fl/fl^* conditional knockouts are microscopically distinct from those that develop in mice lacking β8 integrin in neuroepithelial cells (Fig. 2); (iii) Nrp1 and β8 integrin form intercellular/trans protein complexes and interact genetically to promote adhesion between neuroepithelial cells and endothelial cells in the developing brain (Figs 3,4); (iv) in contrast to mice lacking TGFβR2 or β8 integrin, Nrp1 conditional knockouts display elevated levels of phosphorylated Smads (Figs 5,6; Fig. S9), and (v) Nrp1-dependent defects in Smad signaling and actin cytoskeletal dynamics are detected in cultured endothelial cells (Fig. 7). Collectively, these data identify a paracrine signaling pathway that couples neuroepithelial cells to cerebral blood vessels to balance levels of TGFβ signaling in endothelial cells and control sprouting angiogenesis (Fig. 8).

Alterations in Smad phosphorylation in β8 integrin, TGFβR2, and Nrp1 mutant mice suggest that these proteins functions at distinct, yet interconnected nodes in the TGFβ activation and signaling pathway. αvβ8 integrin is crucial for promoting TGFβ signaling via Smads by adhesion to latent TGFβs in the ECM and activating canonical receptor signaling in endothelial cells. Cell type-specific deletion of integrin expression in the neuroepithelium or TGFβR2 in endothelial cells leads to a major decrease in Smad phosphorylation. Unexpectedly, deletion of Nrp1 in endothelial cells results in increased levels of phosphorylated Smad3 and Smad1/5/8, revealing that Nrp1 acts to suppress TGFβ signaling in endothelial cells. Collectively, these results reveal that a precise balance of TGFβ signaling is essential for normal control of angiogenesis, with abnormally high or low levels of Smad3 activation in endothelial cells leading to similar defects in blood vessel sprouting and brain hemorrhage. Our results differ from other reports showing that Nrp1 promotes canonical TGFβ signaling in non-endothelial cells (Glinka and Prud'homme, 2008; Glinka et al., 2011), indicating cell-type specificity for Nrp1-TGFβ signaling, perhaps resulting from functional connections with β8 integrin in the brain. Along these lines, in cancer cells Nrp1 differentially impacts TGFβ versus bone morphogenetic protein (BMP) signaling via Smads, with RNAi-mediated Nrp1 silencing leading to increased levels of pSmad1/5/8 and diminished levels of pSmad3 (Cao et al., 2010). These data suggest that Nrp1 might differentially modulate TGFβ and BMP signaling in endothelial cells, perhaps by altering the balance of receptor dimers and/or impacting ligand-receptor affinities. Indeed, a recent study reported that Nrp1 suppresses TGFβ signaling via Alk1 and Alk5 in endothelial tip cells to modulate sprouting angiogenesis (Aspalter et al., 2015).

Although TGFβR2 dimerizes with different type 1 receptors, the brain vascular pathologies in Nrp1 mutant mice are most likely a result of defective signaling via the TGFβR2/Alk5 complex. We have reported that selective ablation of Alk5, but not Alk1, phenocopies brain vascular pathologies in TGFβR2 mutants (Nguyen et al., 2011). Although our data demonstrate that Nrp1 suppresses canonical TGFβ signaling, it remains possible that the brain vascular pathologies are also due, in part, to defects in additional Smad-independent signaling effectors. TGFβ receptors activate non-canonical signaling proteins including Cdc42 (Davis and Bayless, 2003; Edlund et al., 2002) and components of the Par protein complex (Bose and Wrana, 2006; Feigin and Muthuswamy, 2009) that control cell polarity and cytoskeletal dynamics. Indeed, our data reveal that Nrp1 regulates actin cytoskeletal dynamics in cultured endothelial cells, and *Nrp1^−/−^* endothelial tip cells display defects in actin-rich filopodia *in vivo*.

β8 integrin is expressed primarily in the developing neuroepithelium, with integrin adhesion to latent TGFβs in the ECM serving as a major pathway for TGFβ activation and signaling *in vivo* (Yang et al., 2007). Nrp1 is robustly expressed in cerebral endothelial cells and at lower levels in the neuroepithelium. Cell type-specific knockout models reveal that endothelial cell-expressed Nrp1 plays a predominant role over Nrp1 expressed in the neuroepithelium (Fantin et al., 2013), which is consistent with our *Alk1-Cre* results. However, our co-immunoprecipitation data also reveal that Nrp1 in the neuroepithelium facilitates the formation of trans interactions between neuroepithelial-expressed αvβ8 integrin and Nrp1 in the endothelium (Fig. 3), which likely affects TGFβ activation and signaling. It remains unclear why angiogenesis pathologies develop primarily in the brains of *Alk1-Cre* conditional knockouts, as the endogenous *Alk1* promoter is active in endothelial cells of multiple organs (Nguyen et al., 2011), and Nrp1 and TGFβ receptors are reportedly expressed in multiple non-neural vascular beds (Iseki et al., 1995). In the developing brain β8 integrin and Nrp1 are obviously crucial components of the latent TGFβ activation and signaling pathway, with loss of either component leading to overlapping angiogenesis pathologies. Perhaps in non-neural tissues other TGFβ family members, for example BMPs, compensate for loss of Nrp1 or TGFβ receptors in endothelial cells. Nonetheless, in the embryonic brain cooperative interactions between β8 integrin and Nrp1 are crucial for proper angiogenesis, and it will be interesting to determine if vascular-related developmental brain disorders are linked to defects in this paracrine adhesion and signaling axis.

## MATERIALS AND METHODS

### Experimental mice

All animal procedures were conducted under Institutional Animal Care and Use Committee-approved protocols. Generation of *Alk1-Cre* and *Tgfbr2^fl/fl^* mice has been detailed elsewhere (Chytil et al., 2002; Nguyen et al., 2011). The *Nrp1^fl/fl^* strain (Gu et al., 2003) was purchased from Jackson Laboratories. Details for generating *Nestin-Cre;β8^fl/fl^* conditional knockouts, *Nestin-Cre;αv^fl/fl^* conditional knockouts, and *β8^−/−^* whole body knockouts have been reported previously (McCarty et al., 2005b; Mobley et al., 2009; Proctor et al., 2005; Lee et al., 2015). The various genetically engineered mice were bred on a mixed genetic background (C57BL6/129S4) and occasionally mated with FVB mice to maintain hybrid vigor. Genotypes of all control and mutant mice were determined using PCR and genomic DNA-based methods. Embryo staging involved timed mating, with noon on the plug date defined as E0.5.

### Zebrafish experiments

All zebrafish embryos were injected at the one-cell stage with 2 ng *p53* MO (GCGCCATTGCTTTGCAAGAATTG) (Robu et al., 2007) as well as combinations of 0.67 ng *itgb8* ATG MO (ATGCAGGAAGTCATAGCAGCTTGA), 0.67 ng *nrp1a* ATG MO (GAATCCTGGAGTTCGGAGTGCGGAA) (Lee et al., 2002), 1.33 ng *itgβ8* SB e2i2 MO (GCGCTCTGGCATACATTACCTCCTG) (Liu et al., 2012) and 1.33 ng *nrp1a* SB e2i3 (AATGTTTTTTCCTTACCCGTTTTGA) (Dell et al., 2013). All MOs were purchased from Gene Tools, LLC. At 24 h post-fertilization, embryos were scored for survival/necrosis and the survivors were treated with 1× PTU in E3 to prevent pigment formation and enable visualization of the brain. Hemorrhages were observed microscopically between 3 and 4 days post-fertilization. Individual hemorrhages were counted once even if they persisted over multiple days. Only embryos with robust circulation were scored. Statistical analysis of hemorrhage rate was performed by N−1 two-proportion test. Genetic synergy was analyzed by comparing the rate of hemorrhage in double injected embryos with the additive rate according to the formula: *itgb8* only+*nrp1a* only/average of the two totals.

### Immunoblotting and immunofluorescence

Embryonic and neonatal brain regions were lysed in 50 mM Tris, pH 7.4, 150 mM NaCl, 1% NP40, 1 mM EDTA containing a cocktail of protease and phosphatase inhibitors (Roche). Detergent-soluble lysates were resolved by SDS-PAGE and then immunoblotted with anti-integrin rabbit polyclonal antibodies at 1:3000 as described previously (McCarty et al., 2005a,b; Mobley et al., 2009; Reyes et al., 2013; Tchaicha et al., 2010). The HRP-conjugated mouse anti-rabbit IgG used for immunoblotting was purchased from Jackson ImmunoResearch (1:1000; Jackson ImmunoResearch, cat. #211-035-109).

Embryos were fixed in cold 4% PFA/PBS for 12-16 h and then embedded in paraffin or agarose and sectioned. The following primary antibodies used for immunofluorescence were purchased from commercial sources: rabbit anti-laminin (1:300; Sigma, cat. #L9393), rat anti-CD31 (1:100; BD Pharmingen, cat. #55370), rabbit anti-NG2 (1:250; EMD Millipore, cat. #AB5320), goat anti-rat Nrp1 (1:100; R&D Systems, cat. #AF566), rabbit anti-Erk1/2 (pThr202/pTyr204; 1:1000; Cell Signaling Technologies, cat. #9101), anti-pSmad3 (pSer423/425; 1:200; Abcam, cat. #ab52903), pSmad1/5/8 (pSer463/465; 1:100; Cell Signaling Technologies, cat. #9511S), rabbit anti-total Smad2/3 (1:100; Cell Signaling Technologies, cat. #3102S) and chicken anti-Nestin (1:500; Neuromics, cat. #CH23001). The anti-β8 integrin polyclonal antibody has been described elsewhere (Jung et al., 2011). Alexa Fluor 488-conjugated Isolectin B4 was purchased from Life Technologies (1:500; cat. #I21411). Commercial antibodies used for immunoblotting include rabbit anti-actin (1:1000; Sigma, cat. #A2066), goat anti-rat Nrp1 (1:1000; R&D Systems, cat. #AF566), goat anti-human Nrp1(1:1000; R&D Systems, cat. #sc-7239), mouse anti-myc (1:3000; Invitrogen, cat. #R950-25) and rabbit anti-TGFβR2 (1:1000; Santa Cruz Biotech, cat. #sc-1700). Secondary antibodies include biotinylated swine anti-rabbit IgG (1:250; DAKO, cat. #E0353), biotinylated rabbit anti-rat IgG (1:250; Vector Laboratories, cat. #BA-4000), biotinylated rabbit anti-goat IgG (1:250; Jackson ImmunoResearch, cat. #305-005-045), and goat anti-rabbit Alexa Fluor 488 IgG (1:500; Jackson ImmunoResearch, cat. #111-545-144). Embryo sections were then analyzed using a Zeiss Axio Imager Z1 microscope. To quantify pSmad3 in CD31^+^ endothelial cells *in vivo*, ratios of the total fluorescence intensity (total intensity of pSmad3/total intensity of CD31) was determined in representative regions of the ganglionic eminence and thalamus (*n*=3 images per region) in control and knockout brain sections (100-150 µm, *n*=3 samples per genotype) prepared with a vibratome. Brain sections were analyzed using a Zeiss confocal microscope.

### Cell culture systems and immunoprecipitation

HUVECs and growth media were purchased from ScienCell. HEK-293 and bEND.3 cells were purchased from ATCC. Serum-starved HUVECs were incubated with TGFβ1 (5 ng/ml) for varying times at 37°C. The pGIPZ lentiviral vectors expressing shRNAs targeting mouse or human Nrp1 were purchased from Dharmacon. To quantify cell adhesion, HUVECs were plated on dishes coated with collagen I (Corning) and stained with crystal violet. Alternatively, adherent HUVECs were fixed, permeabilized, and labeled with Texas Red-conjugated Phalloidin (1:500; Thermo Fisher Scientific, cat. T7471). All HUVECs were analyzed prior to passage 8.

Co-immunoprecipitation experiments to test for cis versus trans interactions between Nrp1 and β8 integrin were performed in HEK-293T cells. V5-tagged human β8 integrin in pcDNA3.1A, full-length rat Nrp1 in pcDNA3.1A, or full-length human NRP1 in pcDNA3.1 were forcibly expressed in HEK-293T cells using Effectene (Qiagen) according to manufacturers' instructions. Twenty-four hours after transfection cells were trypsinized, mixed in various combinations, and co-cultured for an additional 48 h. Detergent-soluble lysates were prepared and immunoprecipitated with anti-V5 antibodies. Antibodies used to distinguish human versus rat Nrp1 were goat anti-rat Nrp1 (1:1000; R&D Systems, cat. #AF566) and goat anti-human NRP1 (1:1000; Santa Cruz Biotechnology, cat. #sc-7239). Alternatively, Nrp1 mutant constructs with various deletions in the extracellular or cytoplasmic domains in pMT21 or pcDNA3.1A mammalian expression plasmids were generated by site-directed mutagenesis. HEK-293 cells were transfected with mammalian expression plasmids using Effectene and lysed in RIPA buffer containing phosphatase and protease inhibitor cocktails (Roche). Plasmids encoding V5-tagged β8 integrin and wild-type rat Nrp1 have been described elsewhere (Gu et al., 2002; Tchaicha et al., 2011).

For cell spreading assays acid-washed coverslips were coated with 10 µg/ml of fibronectin (Millipore) or 5 µg/ml collagen IV (Sigma) in PBS for 1 h at 37°C. Cells were added to coverslips, incubated at 37°C, and then permeabilized with 0.1% Triton X-100 at room temperature for an additional 10 min. Staining was performed with Texas Red-Phalloidin (Life Technologies) and NucBlue Live Cell Stain ReadyProbes (Life Technologies) to visualize the actin cytoskeleton and nuclei, respectively. Statistical analyses were performed using Minitab. Within the same set of images, population analysis of cells with actin accumulation was performed using ImageJ (National Institutes of Health). For quantitative analysis, background corrected images were thresholded to measure intensity of each individual image. Actin accumulation was determined using the masking tool and image statistics tools available in ImageJ. Areas of each individual cell with actin accumulation were normalized to total cell area. Mann–Whitney non-parametric analysis was performed when comparing cells expressing control or *Nrp1* shRNAs.
